# Timing of Antimicrobial Therapy after Identification of Ventilator-Associated Condition Is Not Associated with Mortality in Patients with Ventilator-Associated Pneumonia: A Cohort Study

**DOI:** 10.1371/journal.pone.0097575

**Published:** 2014-05-19

**Authors:** André C. K. B. Amaral, Michael W. Holder

**Affiliations:** 1 Department of Critical Care Medicine, Sunnybrook Health Sciences Centre, Toronto, Ontario, Canada; 2 Interdepartmental Division of Critical Care Medicine, University of Toronto, Toronto, Ontario, Canada; 3 School of Medicine, University of Aberdeen, Aberdeen, Scotland, United Kingdom; D’or Institute of Research and Education, Brazil

## Abstract

**Purpose:**

Delays in antimicrobial therapy increase mortality in ventilator-associated pneumonia (VAP). The more objective ventilator-associated complications (VAC) are increasingly used for quality reporting. It is unknown if delays in antimicrobial administration, after patients meet VAC criteria, leads to worse outcomes.

**Materials and Methods:**

Cohort of 81 episodes of antimicrobial treatment for VAP. We compared mortality, superinfections and treatment failures conditional on the timing of identification of VAC.

**Results:**

60% of patients with VAC had an identifiable episode at least 48 before the initiation of antimicrobials. Antimicrobial administration after the identification of VAC was not associated with intensive care unit (ICU) mortality (OR 0.71, 95% CI 0.11–4.48, p = 0.701) compared to immediate antimicrobial administration. Similarly, the risk of treatment failure or superinfection was not affected by the timing of administration of antimicrobials in VAC (HR 0.95, 95% CI 0.42–2.19, p = 0.914).

**Conclusions:**

We observed no signal of harm associated with the timing to initiate antimicrobials after the identification of a VAC. The identification of VAC should not lead clinicians to start antimicrobials before a diagnosis of VAP can be established.

## Introduction

Ventilator-associated pneumonia (VAP) is one of most common nosocomial infections in critically ill patients [1]. Medical societies and government advocate for the surveillance and public reporting of VAPs as a quality marker of ICU care. However VAPs are difficult to diagnose, current definitions being neither specific or sensitive [2]. In fact, in a recent survey of US hospitals the classification of standardized cases as VAP/non-VAP was almost random among experienced raters, which may explain differences in VAP reporting that are unrelated to quality of care [3]. Therefore, to improve reliability of the definitions, members from several organizations (American Association of Critical Care Nurses, the American College of Chest Physicians, the American Thoracic Society, the Society for Critical Care Medicine, the Association for Professionals in Infection Control and Epidemiology, the Council of State and Territorial Epidemiologists, the Infectious Diseases Society of America, and the Society for Healthcare Epidemiology of America) collaborated to create a new set of definitions that may reduce variability and facilitate inter-hospital comparisons [4]. It is intended for future national surveillance and potentially as a pay-for-performance metric [4].

The new definitions focus on ventilator-associated conditions (VAC) [5], and are based on observational data suggesting an association between worsening ventilatory parameters and mortality [6]. In a recent multicentric study improvements in compliance with VAP prevention recommendations was associated with reduced episodes of VAC [7]. VACs are further categorized into infection-related ventilator-associated condition (IVAC) when an antimicrobial is administered and new VAP definitions are also provided.

As with many public reported quality metrics there is a risk for changes in practice that do not improve patient care [8], [9], or that allow subconscious or conscious gaming of the system [10]. Although the new definitions are intended for surveillance only, and not for clinical use, clinicians will be aware of them and two potential opposing risks exist: (1) early use of antimicrobials for patients with VAC, based on the recommendation from guidelines that antimicrobials should be started once there is clinical suspicion for VAP [11], which could lead to antimicrobial overutilization [12] and diagnostic errors [13]; or (2) withholding of antibiotics for patients with potential VAP to avoid their classification as IVAC, which could lead to delays in appropriate treatment for patients with VAP. Therefore clinicians may benefit from reassurance that the identification of a VAC does not require initiation of antimicrobials.

We designed this study to test the hypothesis that delayed antimicrobial administration, defined as antimicrobials administered after patients met VAC criteria, is associated with worse outcomes in a cohort of patients that ultimately developed VAP. We did not try to answer the more complex question whether gaming of the system may occur and it’s possible impacts on patient outcomes.

## Materials and Methods

### Study Design and Setting

Our study comprised a retrospective cohort of critically ill patients in a 20-bed intensive care unit (ICU) in an academic hospital in Ontario, Canada. This ICU works with a closed-model of physician staffing, and has an antimicrobial stewardship program led by dedicated pharmacists and infectious disease specialists. The ICU team is responsible for decisions regarding initiation of antimicrobials. Once a patient is started on broad-spectrum antimicrobials for VAP the stewardship program reviews the case after 72 hours and makes suggestions. The Research Ethics Board at Sunnybrook Health Sciences Centre approved this study and waived the need for informed consent (project identification number 194-2012).

### Population

We reviewed all cases of patients who received at least 72 hours of broad-spectrum antimicrobials for a clinical suspicion of VAP during the period of July 2011 to June 2012. We excluded patients who received antimicrobials within the first 72 hours of mechanical ventilation, or who received antimicrobials after being extubated for more than 72 hours. We excluded patients who died within 72 hours of starting a broad spectrum antimicrobial, as these are not captured by the stewardship database.

### Variables and Data Collection

Our primary exposure variable was time of initiation of antimicrobials after the identification of a VAC. We used the current definitions for VAC: an increase in the minimum positive end-expiratory pressure (PEEP) during a 24-hour period of 3 cm H2O or an increase in the minimum FiO2 during a 24-hour period of 20%, after a period of 48 hours of stable ventilator settings [14].

We defined VAP based on the previous definitions, including a radiological report compatible with infection, consolidation or opacities, associated with any 2 of the following: temperature >38°C or <36°C, white blood cell count >10 or <5×10^3^/mm^3^, purulent tracheal secretions or respiratory deterioration, as defined above for VAC [15]. With the exception of purulent tracheal secretions, all variables are objectively defined. To avoid ascertainment bias we also used an objective definition for purulent tracheal secretions: episodes of suctioning are charted in the nursing notes with frequency, a semi-quantitative assessment of quantity (from 1 to 3) and a qualitative description of purulence or not. We added the quantitative value for each episode of suctioning that was described as mucopurulent for all patients daily. We used the median value on day -4 as the cut-off value to define purulent secretions.

We collected data using the day of antimicrobial initiation as the index date (day 0) and we also collected data on the 4 preceding days (days -4 to -1). We calculated changes in mechanical ventilation parameters in comparison to the previous day values. Since the data available to qualify a VAC may take 24 hours to be charted, we considered an antimicrobial initiation at the time of VAC if VAC definition was met on the index date or the day preceding the index date. We considered a delayed antimicrobial initiation if VAC definition was met on days -3 or -2 preceding the index date.

For our secondary objectives we defined a superinfection as a new infection within 14 days after the index date. Because superinfections are a concern for antimicrobial over usage, independent of source of infection, we used 3 definitions to identify superinfections: (1) the initiation of a new broad spectrum antimicrobial guided by culture results taken at least 3 days after the initial antimicrobial was started, excluding cases of de-escalation of therapy, or (2) positive blood cultures for microorganisms that were not covered by the initial antimicrobial or (3) initiation of empirical antimicrobial treatment for *Clostridium difficile*, *Candida sp* or methicillin-resistant *Staphylococcus aureus* (MRSA). We defined failure to treat the initial infection as the initiation of a new antimicrobial with broader spectrum than the antimicrobial started on the index date, after at least 48 hours of treatment, in the absence of positive cultures.

We defined an appropriate antimicrobial for VAP using the American Thoracic Society guidelines [16].

### Statistical Analysis

We described the baseline characteristics of patients with means and standard deviations, medians and interquartile ranges or proportions, as appropriate. We performed univariate analysis using chi-square or Fisher’s exact test for categorical variables and t-test or Wilcoxon’s rank-sum test for continuous variables. We used logistic regression to model the main effects of a delay in antimicrobial initiation on mortality, while adjusting for confounders. For the analysis of mortality we only included data on the first episode of VAC for patients that had more than one episode. We coded PaO_2_/FiO_2_ ratios as hypoxemia for values below 300 and temperature as fever for values above 37.8°C. We included variables that were associated with mortality in the univariate analysis with a p<0.15 in a stepwise forward regression model with a variable selection threshold of 0.10. For our secondary outcome, we combined superinfection and failure to treat as the outcome variable and used a Cox Proportional Hazards model to account for the censoring effect of discharge to a ward and for the competing risk of death, according to the method of Lunn and McNeil [17].

## Results

We identified 117 episodes of VAP in the antimicrobial stewardship database. We excluded 26 episodes where antimicrobials were started before 72 hours of mechanical ventilation or after 72 hours of extubation and 10 episodes as the medical records were unavailable at the time of the study. We analyzed 81 episodes in 78 unique patients. Patient characteristics of each episode are shown in table 1. Reflecting the patient population at our center, most patients were male and were admitted with trauma or a medical condition as their primary diagnosis. The median length of stay before initiation of an antimicrobial for clinical suspicion of VAP was 7 days (4 to 13) and the median sequential organ failure assessment (SOFA) score on the Index Day was 4 (2 to 6). ICU mortality was 20% and hospital mortality was 31%. 59% of patients had positive cultures, with *Pseudomonas aeruginosa*, *Haemophilus influenza* and *Staphylococcus aureus* being the 3 most common organisms. 90% of patients used appropriate antimicrobial therapy, 12% failed their initial therapeutic regimen and 42% developed a superinfection within 14 days.

**Table 1:** Episode characteristics.

	All patients (81)	VAC(45)	No VAC(36)
Age, Mean (SD)	58 (22)	54 (24)	62 (19)^a^
Sex, Male (%)	84%	82%	86%
Admission Diagnosis			
Trauma	56%	60%	53%
Medical	26%	31%	22%
Surgical	9%	9%	8%
Other	9%	0%	17%
ICU LOS Before Index Day,Median (IQR)	7.2 (4.3–13.2)	6.5 (4.4–10.4)	8.3 (4.3–16.3)
SOFA Score on Index Day,Median (IQR)	4 (2–6)	3 (2–6)	4 (1–6)
Lactate, mmol/L, on Index Day,Median (IQR)	1.3 (1–1.7)	1.3 (1–1.7)	1.3 (1–1.7)
Nor-epinephrine equivalents, mcg/min,on Index Day, Median (IQR)	0 (0–8.3)	0 (0–8)	0 (0–9.2)
Patients using vasopressors on Index Day, %	37%	38%	36%
PaO2/FiO2 on Index Day,Mean (SD)	268 (88)	239 (84)	305 (80)^b^
PEEP on Index Day, cm H20,Median (IQR)	8 (5–10)	8 (5–10)	5 (5–9)^a^
FiO2 on Index Day,Median (IQR)	0.4 (0.3–0.5)	0.4 (0.35–0.5)	0.35 (0.3–0.4)^a^
Minute Ventilation on Index Day,L/min, Mean (SD)	15.3 (4.4)	15.5 (4.5)	15.1 (4.3)
Platelets, x10^3^/mm^3^, on Index Day,Median (IQR)	278 (181–423)	267 (176–393)	289 (189–483)
WBC on Index Day, x10^3^/mm^3^,Mean (SD)	16.6 (6.6)	14.9 (5.9)	18.9 (6.8)^a^
Creatinine, mmol/L, on Index Day,Median (IQR)	70 (51–116)	67 (53–100)	80 (50–139)^a^
Urine Output, ml/24 hours, on Index Day,Median (IQR)	2105 (1275–3180)	2155 (1225–3790)	2097 (1295–3010)
Temperature, °C, on Index Day,Mean (SD)	38.4 (0.9)	38.3 (0.9)	38.4 (0.9)
Length of Mechanical Ventilation,hours, Median (IQR)	419 (274–655)	367 (268–649)	504 (291–673)
ICU LOS, days, Median (IQR)	24 (15–35)	26 (15–35)	23 (14–34)
Hospital LOS, days, Median (IQR)	45 (32–61)	49 (32–63)	38 (31–58)
ICU Mortality, % (95% CI)	20 (11–29)	20 (8–32)	19 (6–33)
Hospital Mortality, % (95% CI)	31 (21–41)	29 (15–43)	33 (17–49)
Positive Cultures*			
Pseudomonas aeruginosa	10%	7%	14%
Haemophilus influenza	11%	13%	8%
Staphylococcus aureus	7%	9%	5%
Klebsiella oxytoca	6%	4%	8%
Klebsiella pneumonia	6%	2%	11%
Escherichia coli	5%	7%	3%
Serratia marcescens	4%	0%	8%
Other	10%	11%	8%
No Positive Cultures	41%	47%	35%
Initial Antimicrobial			
Piperacillin/Tazobactam	62%	67%	56%
Ciprofloxacin	17%	8%	28%
Ceftriaxone	7%	11%	3%
Meropenem	6%	4%	8%
Other	8%	10%	5%
Appropriateness of initial Antimicrobial,% (95% CI)	90 (83–97)	87 (76–97)	94 (87–100)
Failure of Initial Treatment, % (95% CI)	12 (5–20)	13 (3–24)	11 (3–22)
Superinfection, % (95% CI)	42 (31–53)	44 (29–59)	39 (22–56)

a p<0.05;

b p 0.01.

Forty-five episodes of antimicrobial administration for suspected VAP fulfilled criteria for VAC. Patients without VAC were older, had better PaO_2_/FiO_2_ ratios, and higher creatinine and white blood cell count (WBC) than patients with VAC (Table 1). Sixty percent of patients developed VAC before the Index Day or Day -1 and were considered in the delayed administration group. Patients with delayed administration of antimicrobials after the identification of VAC were similar in baseline characteristics and crude outcomes to those without delays, with the exception of older age for patients with VAC who received a delayed administration of antimicrobials (table 2).

**Table 2:** Comparisons between patients with and without delays after the identification of a VAC.

	VAC (45)	
	No Delay (18)	Delay (27)
Age, Mean (SD)	47 (27)	59 (21)^a^
Sex, Male (%)	83%	81%
ICU LOS Before Index Day,Median (IQR)	8 (6–10)	6 (4–11)
SOFA Score on Index Day,Median (IQR)	3 (1–5)	4 (2–7)
Lactate, mmol/L, on Index Day,Median (IQR)	1.1 (1.0–2.3)	1.3 (1.0–1.6)
Nor-epinephrine equivalents, mcg/min,on Index Day, Median (IQR)	0 (0–8)	0 (0–9)
Patients using vasopressors on Index Day, %	39%	37%
PaO2/FiO2 on Index Day,Mean (SD)	244 (84)	236 (86)
Minute Ventilation, L/min,on Index Day, Mean (SD)	14.0 (3.4)	15.8 (4.8)
WBC on Index Day, x10^3^/mm^3^,Mean (SD)	15.7 (6.0)	14.4 (6.0)
Temperature, °C, on Index Day,Mean (SD)	38.4 (1.0)	38.2 (0.9)
Length of Mechanical Ventilation,hours, Median (IQR)	379 (275–550)	365 (222–706)
ICU LOS, days,Median (IQR)	22 (15–32)	27 (14–35)
ICU Mortality, % (95% CI)	17 (0–36)	22 (5–39)
Failure of initial treatment, % (95% CI)	11 (0–27)	15 (0–29)
Superinfection, % (95% CI)	39 (14–64)	48 (28–68)

a p<0.05.

### Mortality

In patients with VAC, age (OR 1.06, 95% CI 1.01–1.12, p = 0.009), SOFA scores on the index day (OR 1.33, 95% CI 1.04–1.71, p = 0.024), and nor-epinephrine equivalents on the index day (OR 1.07, 95% CI 1.01–1.14, p = 0.019) were positively associated with ICU mortality. Fever on index day (OR 0.12, 95% CI 0.02–0.61, p = 0.010) was negatively associated with ICU mortality. Delayed antimicrobial administration after VAC was not associated with ICU mortality in unadjusted (RR: 0.75, 95% CI 0.11–3.96, p = 0.721) and adjusted analysis (OR 0.71, 95% CI 0.11–4.48, p = 0.701).

### Treatment Failure or Superinfection

The risk of treatment failure or superinfection was not affected by delayed administration of antibiotics after VAC (HR 0.95, 95% CI 0.42–2.19, p = 0.914).

## Discussion

In a single-centre cohort of patients who received antimicrobials for VAP we observed that 60% of those who presented with VAC had at least 48 hours between the identification of VAC and the initiation of antimicrobial therapy. We observed no association between the timing of identification of VAC and mortality, superinfection or treatment failure.

Our findings are re-assuring, as it suggests that clinicians may wait for a more clear diagnosis of infection when faced with changes in ventilatory parameters after a period of stability. This is an important finding, as previous observational data, based on the association between modification of antimicrobial therapy after the availability of culture results and mortality [18]–[23], provide indirect evidence of harm for delays in antimicrobial therapy for VAP. While more recent evidence suggests that a “watch and wait” approach for antimicrobials in VAP may have a mortality benefit [24], clinicians may still be tempted to extrapolate the observational data and guidelines recommendations to patients with respiratory deterioration.

Our findings are expected, as the absolute mortality differences observed in the observational studies are probably overestimated, varying from 31 to 53% [19], [25], [26], compared to the 5% attributable mortality of VAP [27]. Hence, it would be unlikely that VACs could benefit from early antimicrobials. Furthermore, most of the previous reports actually define delays as changes in antimicrobial therapy due to inappropriate empirical coverage, which is different than delaying therapy to wait for culture results. Patients that have an initial inappropriate empirical therapy are more likely to host antimicrobial resistant organisms, such as *Pseudomonas* and *Acinetobacter* [18], [28], which are associated with a higher mortality [29]. The percentage of patients receiving inappropriate therapy was very high in the previous studies, varying from 30 to 68% [18]–[20], [25], as compared to 10% in our cohort.

Our findings are important for clinicians, as we observed that delays in antimicrobial administration are frequent in patients with VAC and should not prompt the initiation of antimicrobials until a more firm diagnosis of IVAC or VAP is established. Since the diagnosis of VAP has a low reliability [30] and guidelines recommend the initiation of antimicrobials before the availability of culture results [11], it is possible to overtreat patients with two main consequences, (1) the appearance of antimicrobial-resistant organisms and *Clostridium difficile* and (2) cognitive errors, leading to missed diagnosis of other clinical entities that may require further investigations and treatment [31]. This strategy is based on generalization of the association between delays in antimicrobial administration and mortality in septic shock [32] and on one small trial of early empirical versus delayed, culture-guided, antimicrobials in trauma patients [33], which reported no benefits of delayed, culture-guided therapy.

Our study has several limitations. We have a small number of events, which may over fit our data. Although the rule of thumb suggests that there should be 5 to 10 events per covariate, simulations suggest that this rule can be relaxed for adjustments, especially when the associations remain significant [34]. However, a type II error may still be present and we would not discard the possibility that the lack of harm observed from delaying antimicrobials for patients with VAC is due to overfitting. Furthermore, we have no information on patients that died within the first 72 hours of antimicrobial therapy and it is possible that this population may benefit from earlier administration of antimicrobials. We also do not have data on patients that developed similar clinical syndromes, but never received antimicrobials. Having data on this group would allow us to make more strong inferences to the early use of antimicrobials after the development of clinical deterioration. This study was also not designed to answer the other potential problem with a new quality indicator, which is to avoid or delay antimicrobial therapy for patients with VAP, thus decreasing the incidence of IVACs at the institutional level.

## Conclusion

We observed no signal of harm in delaying antimicrobials after the identification of a VAC. Our findings are important to provide clinicians with the confidence to wait for other clinical signs of infection in patients with worsening respiratory function. Due to the limitations of the study design and sample size these findings should be interpreted as hypothesis generating, as we also did not observe harm due to the early administration of antimicrobials for VAC.
